# PXR-Mediated Upregulation of CYP3A Expression by Herb Compound Praeruptorin C from *Peucedanum praeruptorum *Dunn

**DOI:** 10.1155/2013/156574

**Published:** 2013-11-26

**Authors:** Ling Huang, Qian Wu, Yu-Hua Li, Yi-Tao Wang, Hui-Chang Bi

**Affiliations:** ^1^Laboratory of Drug Metabolism and Pharmacokinetics, School of Pharmaceutical Sciences, Sun Yat-sen University, 132 Waihuan Dong Road, Guangzhou 510006, China; ^2^School of Pharmaceutical Sciences, Hainan Medical University, Haikou 571010, China; ^3^Institute of Chinese Medical Sciences, Macau University, Macau 999078, China

## Abstract

We recently reported that Praeruptorin C effectively transactivated the mRNA, protein expression, and catalytic activity of CYP3A4 via the CAR-mediated pathway, but whether and how PC could affect the expression and catalytic activity of CYP3A4 via PXR pathway remains unknown. Therefore, in this study, the effect of PC on the *CYP3A* gene expression was investigated in mice primary hepatocytes after knockdown of PXR by transient transfection of PXR siRNA, and the gene expression, protein expression, and catalytic activity of CYP3A4 in the LS174T cells with PXR overexpression were determined by real-time PCR, western blot analysis, and LC-MS/MS-based CYP3A4 substrate assay, respectively. We found that the level of *CYP3a11* gene expression in mouse primary hepatocytes was significantly increased by praeruptorin C, but such an induction was suppressed after knockdown of pregnane X receptor by its siRNA. In PXR-overexpressed LS174T cells, PC significantly enhanced CYP3A4 mRNA, protein expression, and functional activity through PXR-mediated pathway; conversely, no such increase was found in the untransfected cells. These findings suggest that PC can significantly upregulate CYP3A level via the PXR-mediated pathway, and this should be taken into consideration to predict any potential herb-drug interactions between PC, Qianhu, and the other coadministered drugs.

## 1. Introduction

The root of *Peucedanum praeruptorum *Dunn. (Qianhu) is widely used in traditional Chinese medicine as antitussive, anti-inflammatory, and antiasthma component and as a remedy for angina. In China, Qianhu is an important ingredient in many kinds of famous traditional Chinese medicine preparations. Most traditional Chinese medicine prescriptions for antitussive contain Qianhu. In recent years, pharmacological evaluations have also revealed a wide variety of activities of Qianhu, including hypotensive [1], coronary dilatory [2], and myocardial dysfunction [3]. Praeruptorin C (PC) is the major active constituent isolated from *Peucedanum praeruptorum* Dunn. PC has been proved to possess multiple pharmacological activities such as prevention and treatment of vascular hyperplastic disease [4], relaxation of the smooth muscle of tracheas and pulmonary arteries [1], relaxation of coronary artery, and decreased contractility activity in left atria [5]. For the increasing wide use of Qianhu and its active component PC in the clinical practice, potential of clinical herb-drug interactions is strikingly increased, and thus, it is important to predict these potential herb-drug interactions.

The underlying mechanisms for most reported herb-drug interactions have not been clearly elucidated, but induction or inhibition of cytochrome P450 (CYP) enzymes is one of the most important risk factors for drug-drug interactions (DDIs). CYP3A4 is responsible for metabolic conversion of more than 50% of the currently clinical drugs to more polar metabolites for easier excretion [6]. Induction or inhibition of CYP3A4 by xenobiotics contributes to the pronounced interindividual variability of its expression and often results in clinically relevant DDIs or herb-drug interactions [7–9]. Clinically and preclinically relevant interactions have been hugely reported between herbs and drugs such as St. John's wort, pomelo, and grapefruit juice, and induction or inhibition of CYP3A4 by xenobiotics often results in these herb-drug interactions [10, 11]. Therefore, CYP3A4-related DDIs have significant clinical impacts.

In recent years, important advances have been made in mechanisms involved in induction or inhibition of CYP3A4. A family of ligand-activated transcription factors, known as nuclear receptors (NRs), has been identified as mediators of drug-induced expression of CYP3A4. Among them, the pregnane X receptor (PXR, NR1i2) and the constitutive androstane receptor (CAR, NR1i3) are the mainly mediator of CYP3A4 [12–14].

To date, pregnane X receptor- (PXR-) mediated CYP3A4 induction has been well studied. PXR can be activated by a wide variety of small, hydrophobic endogenous and exogenous compounds. A number of naturally occurring compounds from herbs such as St John's wort [15], Ginkgo (*Ginkgo biloba*) [16, 17], Gugulipid (*Commiphora mukul*) [18], Wu Wei Zi (*Schisandra chinensis*), Licorice (*Glycyrrhiza uralensis*) [19], and Dan Shen (*Salvia miltiorrhiza*) [20] have been reported to activate PXR. Upon activation by a ligand, PXR unites with RXR*α* to bind and transactivate several specific elements, such as the everted repeat with a six-nucleotide spacer (ER6) or a direct repeat with a three-nucleotide spacer (DR3), in the 5′ upstream regulatory region of the *CYP3A4* gene [21, 22], thus play a role in regulating transcription of CYP3A4. Therefore, over 64% pharmaceutical companies in the US have adopted cell-based PXR reporter assays routinely to assess the potential for DDIs due to CYP3A4 inductions [23]. In our most recent studies, we found that the active ingredients PA and PD of Qianhu could upregulate CYP3A4 expression by PXR [24, 25], but whether and how PC could regulate CYP3A4 transcription through PXR pathway remains unclear.

The nuclear hormone receptor CAR is a sister xenobiotic receptor of PXR and plays a pivotal role in the induction of drug metabolism. CAR has been reported to synergistically regulate the transcriptional activity of the *CYP3A4* with PXR [26]. According to our previous results [27], PC induction of CYP3A4 at the transcriptional level could be activated through CAR pathways. PC effectively transactivated the mRNA, protein expression, and catalytic activity of CYP3A4 via the CAR-mediated pathway in human LS174T cells. We also found that CYP3A4 luciferase expression was enhanced through PXR pathway by PC [28]. However, the results of luciferase reporter assay could not fully prove that PC could affect the CYP3A4 expression through PXR nuclear receptor pathway. Further study is needed to investigate whether PC could upregulate CYP3A transcription by activation of PXR pathway and whether PC could affect CYP3A4 activity by the cross-talk mediated effect of PXR and CAR.

Therefore, in this study, regulation of *Cyp3a11* (CYP3A4's homolog in mouse) gene expression by PC through PXR pathway was confirmed in mice primary hepatocytes after knockdown of PXR by transient transfection of PXR siRNA. On the other hand, the gene expression, protein expression, and catalytic activity of CYP3A4 were compared in LS174T cells and PXR overexpressed LS174T cells to further prove the effect of PC on PXR and CYP3A and thus to predict any potential herb-drug interactions between PC, Qianhu, and the coadministered drugs.

## 2. Material and Methods 

### 2.1. Ethics Statement

All procedures of animal experiments, in this study, were in accordance with the Regulations of Experimental Animal Administration issued by the Ministry of Science and Technology of the People's Republic of China. The animal study protocols were approved by the Institutional Animal Care and Use Committee (IACUC) at Sun Yat-sen University (Guangzhou, China) on October 2010, and the approved number was IACUC-01101028.

### 2.2. Animals

Male BALB/c mice (6–8 weeks old) were purchased from Medical Experimental Animal Center of Guangdong Province and kept in a room at 22–24°C with a light/dark cycle of 12/12 h and 55–60% relative humidity in Experimental Animal Center of Sun Yat-sen University. They had free access to standard rodent chow and clean tap water.

### 2.3. Chemicals and Reagents

**Figure 1:**
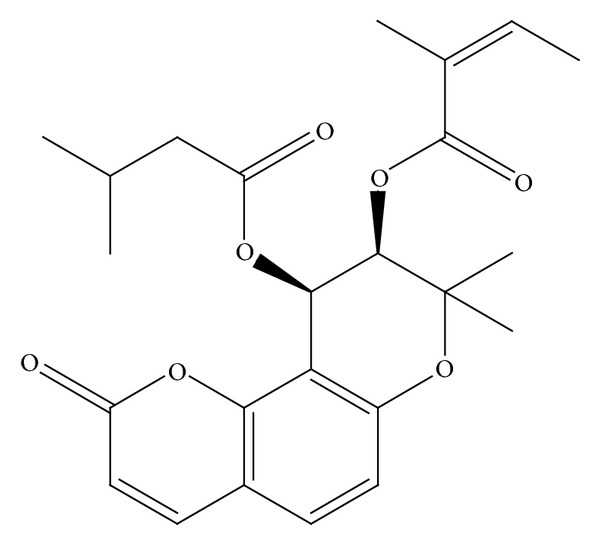
PC physicochemical properties investigated in this study. CAS Number: 73069-27-9. Molecular Formula: C_22_H_22_O_8_.

Praeruptorin C (Figure 1) (purity > 99%) was available from Kui Qing Chemical Company (Tianjin, China). Dimethyl sulfoxide (DMSO), dexamethasone (DEX), nifedipine (NIF), dehydronifedipine (DNIF), and loratadine were purchased from Sigma-Aldrich (St. Louis, MO, USA). The validated siRNA targeted to the PXR gene and nontargeting siRNA as a silencer negative control were purchased from Guangzhou RiboBio Co., Ltd (Guangzhou, China). siRNA Transfection Reagent was purchased from Roche (New Jersey, USA). RNAiso Plus and PrimeScriptTM RT Reagent were obtained from Takara (Kyoto, Japan). The *Cyp3a11*, PXR, CYP3A4, and glyceraldehyde-3-phosphate dehydrogenase (GAPDH) primers used in real-time PCR were synthesized by Takara. Anti-CYP3A4 polyclonal antibody was purchased from Millipore Corporation (Rosemont, IL, USA); anti-Cyp3a11 polyclonal antibody and GADPH antibody were purchased from Cell Signaling Technology (Danvers, MA, USA). Anti-rabbit IG-HRP antibody was purchased from R&D Systems (Minneapolis, MN, USA). SDS-PAGE Gel Preparation Kit was purchased from Beyotime Institute of Biotechnology (Haimen, Jiangsu, China). Plasmocin TM Ant-mpp and complete protease inhibitor cocktail were purchased from Invitrogen (San Diego, CA, USA) and Roche Diagnostics (Basel, Switzerland), respectively. The cytotoxicity of LS174T cells were detected by the 3-(4,5)-dimethylthiahiazo (-z-y1)-3,5-di-phenytetrazoliumromide (MTT) cytotoxicity assay, and PA did not show cytotoxicity in LS174T cells under the maximum dosage (40 *μ*M).

### 2.4. Plasmid

The pSG5-hPXR expression vector was provided generously by Dr. Steven Kliewer (University of Texas Southwestern Medical Center, Dallas, TX, USA) [29]. The pGL3-CYP3A4-XREM luciferase reporter construct containing the basal promoter (−362/+53) with the proximal PXR response element (ER6) and the distal xenobiotic responsive enhancer module (XREM, −7836/−7208) of the CYP3A4 gene 5-flanking region inserted to pGL3-Basic reporter vector was provided generously by Dr. Jeff Staudinger, Department of Pharmacology and Toxicology, University of Kansas, Lawrence, KS, USA [30]. The pRL-TK *R. reniformis* control vector and pSG5-empty vector were obtained from Promega (Madison, WI, USA).

### 2.5. Preparation of Primary Cultures of Mice Hepatocytes

Liver cells were isolated from BALB/c male mice at 6–8 weeks of age by the two-step collagenase perfusion technique previously described [31] with slight modifications [32]. A density of 2-3 × 10^6^ mice primary hepatocyte per g liver tissue with more than 70% cell viability was obtained. Standard culture conditions were as follows: the cells were dispersed in William's medium E containing 10% FBS, insulin (0.5 U/mL), and hepatocytes were seeded into 24-well collagen-coated plate at a density of 1 × 10^5^ cells/well. The hepatocytes anchored to the collagen-precoated plates within 8 h and subsequently formed a monolayer, and only hepatocytes with viability greater than 90% were used for this study. The 24-well collagen-coated plate was maintained at 37°C in 5% CO_2_-humidified incubator.

### 2.6. Transfection of siRNA and Treatment

Mice primary hepatocytes were placed on a 24-well plate at a density of 1 × 10^5^ cells/well. For transfection, hepatocytes were transfected with siRNA targeted to the *mPXR* gene and nontargeting siRNA as a silencer negative control using siRNA transfection reagent (Roche, USA) according to the manufacturer's instructions. For each well, a mixture of siRNA transfection reagent complex was delivered to cells with final concentrations of 50 nM. For monitoring the gene silencing effect, cells were harvested after 72 h, then total RNA was extracted from mice hepatocytes and *mPXR* gene expression was investigated by Q-PCR. Then, the PXR siRNA transfection hepatocytes and negative siRNA transfection hepatocytes were further incubated with 10 *μ*M DEX, 2.5 *μ*M, 10 *μ*M, and 40 *μ*M PC for 72 h. Total RNA was extracted and *Cyp3a11* gene expression was investigated by qPCR. The si-RNA untransfected wild mice primary hepatocytes were exposed to DMSO (0.1%), dexamethasone at 10 *μ*M, PC at 2.5, 10, and 40 *μ*M for 72 h to observe whether PC has the induction effect on mPXR.

### 2.7. Real-Time PCR Analysis of *Cyp3a11* mRNA and m*PXR* mRNA

Total RNA of mice hepatocytes was isolated using Trizol reagent (Invitrogen) according to the manufacturer's instruction. Total RNA was quantified and reversely transcribed into cDNA using PrimeScript RT reagent Kit (Takara, Kyoto, Japan). The primers for *Cyp3a11* mRNA and m*PXR* mRNA detection were designed as described in our previous report [26]. All the PCR reactions were carried out using SYBR Premix Ex TaqTM kit (Takara, Kyoto, Japan) and according to manufacturer's instructions. Amplification was performed in PCR-Capillarys on a Light Cycler 2.0 Real Time Detection System (Roche, Hercules, CA, USA). Amplification of predenatured products was conducted at 94°C for 60 sec, followed by 45 cycles at 95°C for 30 sec, 59°C for 30 sec, 72°C for 30 sec, 95°C for 10 sec, 65°C for 45 sec, and 40°C for 60 Sec. Fold induction values were calculated according to the equation 2^−ΔΔCt^, where ΔCt represents the differences in cycle threshold numbers between the target gene and *GADPH*, and ΔΔCt represents the relative change in the differences between control group and treatment group. The data presented are the mean ± S.E.M of triplicate experiments.

### 2.8. LS174T Cells Culture

LS174T cells (derived from Caucasian colon adenocarcinoma) were purchased from Shanghai Institute for Biological Sciences cell resource center. LS174T cells were maintained in Roswell Park Memorial Institute (RPMI) 1640 medium (Hyclone, Logan, UT, USA) supplemented with 10% fetal bovine serum (FBS) (Hyclone, Logan, UT, USA). Cell lines were cultured at 37°C under a humidified atmosphere of 5% CO_2_.

### 2.9. PXR Expression Plasmid Transient Transfection and Total RNA Isolation

LS174T cells (1.2 × 10^5^ per well) were seeded into 24-well plates, cultured for 24 h, and then were transfected with PXR expression plasmids (300 ng/well). Appropriate cell samples were exposed to PC at a concentration of 2.5, 10, and 40 *μ*M for 48 h. Incubations with 10 *μ*M RIF and DMSO (0.1%) were also included as the controls. Total RNA was isolated using Trizol reagent (Invitrogen) according to the manufacturer's instruction. Total RNA was quantified and reverse transcribed into cDNA using PrimeScriptTM RT reagent Kit (Takara, Kyoto, Japan).

### 2.10. Real-Time PCR Analysis of CYP3A4 mRNA

The primers for *CYP3A4* mRNA detection were designed as described in our previous paper [24]. All the PCR reactions were carried out using SYBR Premix Ex TaqTM kit (Takara, Kyoto, Japan) and according to manufacturer's instructions. Amplification of predenatured products was conducted at 94°C for 60 sec, followed by 45 cycles at 95°C for 30 sec, 58°C for 30 sec, 72°C for 30 sec, 95°C for 10 sec, 65°C for 45 sec, and 40°C for 60 Sec. Fold induction values were calculated according to the equation 2^−ΔΔCt^, where ΔCt represents the differences in cycle threshold numbers between the target gene and **β*-actin*, and ΔΔCt represents the relative change in the differences between control group and treatment group. The effect of PA on* CYP3A4* mRNA levels is presented as fold mRNA expression to vehicle control.

### 2.11. PXR Expression Plasmid Transient Transfection and Western Blotting Analysis

LS174T cells (1.0 × 10^6^ per well) were seeded into 6-well plates, cultivated for 24 h, and then transfected with hPXR expression plasmids (1 *μ*g/well). Appropriate cell samples were exposed to DMSO (0.1%), RIF at 10 *μ*M, and PC at 2.5, 10, and 40 *μ*M for 72 h. All the proteins were extracted by high-speed centrifugation and quantified using Coomassie Protein Assay Kit (Pierce, Rockford, IL,USA). CYP3A4 protein levels were measured by Western blot analysis, and the Western blot analysis was conducted as described in our previous study [27]. The data were expressed as relative folds over vehicle controls.

### 2.12. PXR Expression Plasmid Transient Transfection and Functional Analysis of CYP3A4 Activity

LS174T cells (1.0 × 10^6^ per well) were seeded into 6-well plates and cultivated for 24 h. Cells were transfected or untransfected with hPXR expression plasmids (1 *μ*g/well) and then exposed to PC at a concentration of 2.5, 10, and 40 *μ*M for 72 h. The cells were lysed using radio immunoprecipitation assay (RIPA) buffer plus phenylmethylsulfonyl fluoride (PMSF); total protein was extracted by high-speed centrifugation and quantified using Coomassie Protein Assay Kit (Pierce, Rockford, IL,USA). The protein pretreatment was performed as described in our previous report [27], and the concentration of the nifedipine metabolite was determined using an established LC-MS/MS method [33].

### 2.13. Statistical Analysis

One-way ANOVA followed by Dunnett's multiple comparison post hoc test or unpaired Student's *t*-test was used for statistical analysis of data using SPSS version 13.0 software (SPSS Inc, Chicago, IL, USA). Probability values <0.05 were considered to be statistically significant.

## 3. Results

**Figure 2:**
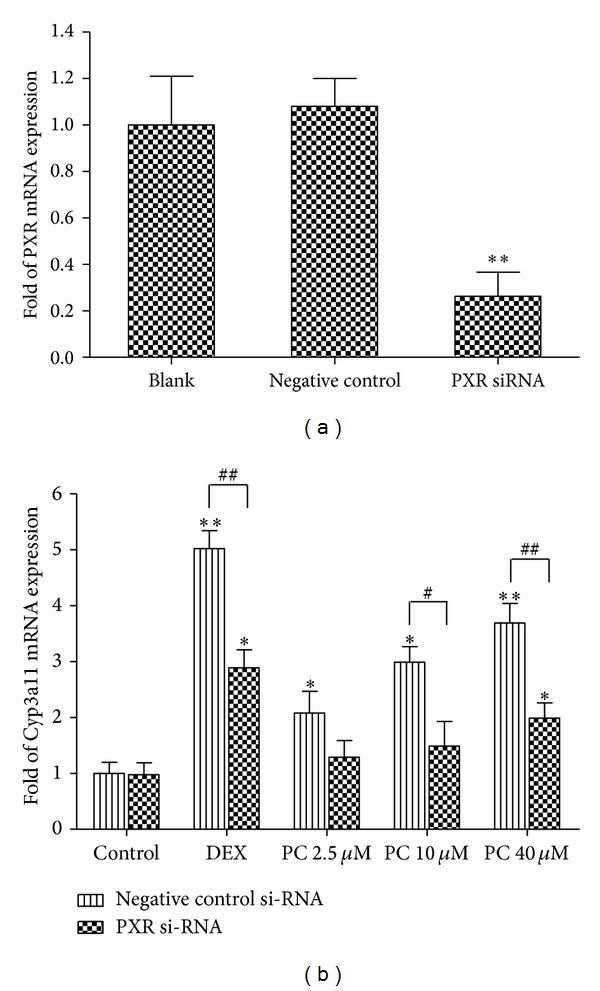
Effects of PC on *Cyp3a11 *mRNA expression in PXR knockdown mice primary hepatocytes cells. (a) Mice primary hepatocyte were transfected with negative control siRNA (50 nM) or PXR siRNAs (50 nM). *PXR *mRNA levels were analyzed by real-time PCR. **P < 0.01. (b) Mice primary hepatocyte were transfected with negative control (50 nM) or PXR siRNAs (50 nM) then were treated with 10**** *μ*M DEX and 2.5, 10, and 40**** *μ*M PC for 72 h, respectively. The *Cyp3a11* mRNA expression was analyzed using real-time quantitative PCR.*P < 0.05, **P < 0.01, for compared with the blank in control siRNA transfected groups, ^#^ *P* < 0.05, ^##^ *P* < 0.01 for comparison between negative control siRNA and PXR siRNA. Values are expressed as mean ± S.E.M (*n* = 3).

The primary hepatocytes used in the study were isolated from wild-type mice and PXR knockdown mice primary hepatocytes were further obtained by transient transfection of PXR siRNA. A density of 2-3 × 10^6^ cells per g liver tissue was obtained. Approximately 3–5 × 10^6^ cells per mouse were harvested with viability and attachment efficiency above 90%. The silencing effects of siRNA transfection on *PXR* gene expression were confirmed by quantitative real-time PCR.  Figure 2(a) shows that the *PXR* mRNA level were significantly decreased to 26% in mice primary hepatocytes after transfection of PXR siRNA at 50 nM. Furthermore, no inhibitory effect on *PXR* gene expression was observed in mice primary hepatocytes after transfection of the negative control siRNA.

The effect of PC on the *Cyp3a11* mRNA levels in mice primary hepatocytes with or without PXR knockdown was measured.  Figure 2(b) shows that significantly enhanced induction of* Cyp3a11 *mRNA expressions by PC were observed in mice primary hepatocytes transfected with the negative control siRNA. After treating the negative control siRNA transfected hepatocytes with PC at 10 *μ*M and 40 *μ*M for 72 h, the level of *Cyp3a11* mRNA was increased to 3.01-fold and 3.69-fold, respectively (*P* < 0.05, *P* < 0.01). However, compared with the PXR unknockdown group, the induction of *Cyp3a11* mRNA expression by PC (10 *μ*M and 40 *μ*M) was significantly suppressed 41.8% and 46.3% when PXR siRNA was transfected (*P* < 0.05).

**Figure 3:**
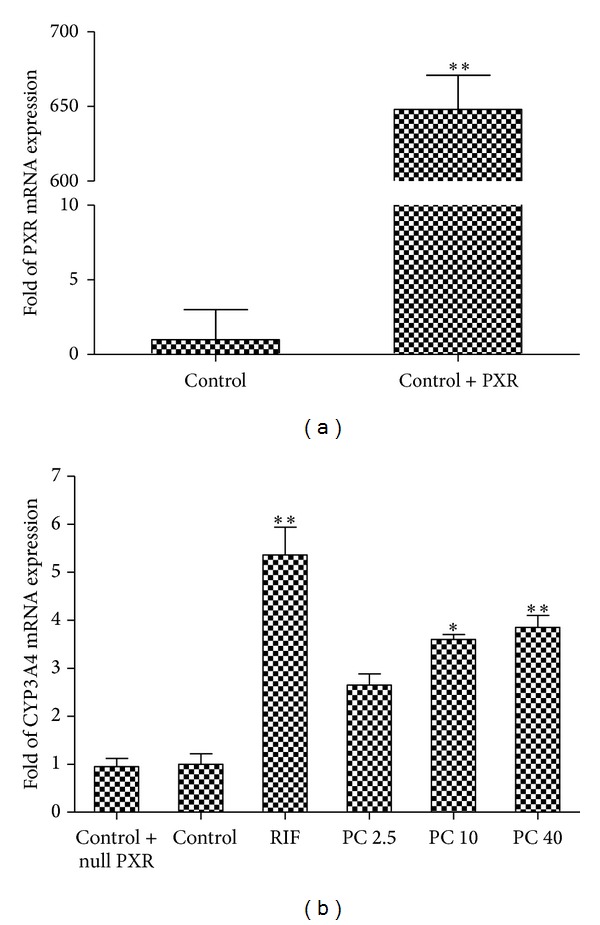
Effects of PC on the expression of *CYP3A4* mRNA in LS174T cells. (a) LS174T cells were transfected with hPXR expression plasmids for 6 h. Total RNA of LS174T cells was isolated, and *hPXR mRNA* levels were analyzed by real-time PCR. The effect of herbal compounds on* hPXR* mRNA levels is presented as fold mRNA expression to control vehicle treated cells. (b) LS174T cells were transfected with hPXR expression plasmids for 6 h. The cells were treated with vehicle control (0.1% DMSO); 10 *μ*M CITCO; and 2.5, 10, and 40 *μ*M PC for 48 h, respectively. The *CYP3A4* mRNA levels were analyzed by real-time PCR. The effect of herbal compounds on* CYP3A4* mRNA levels is presented as fold mRNA expression to control vehicle treated cells. *P < 0.05, **P < 0.01 for comparison with the control groups. Values are expressed as mean ± S.E.M (*n* = 3).

Previously, we found that PC significantly transactivated CYP3A4 reporter gene construct in PXR transiently transfected LS174T cells [27]. But *CYP3A4* mRNA level was not significantly increased in the untransfected LS174T cells after administered PC. In order to determine whether or not PC induces CYP3A4 expression by PXR-mediated pathway, LS174T cells were transfected with pSG5-hPXR expression plasmids, and cells were exposed to PC at 2.5, 10, and 40 *μ*M for 48 h, then the mRNA levels of *PXR* and *CYP3A4* were detected by real-time PCR. As shown in Figure 3(a), compared with the untransfected LS174T cells, the PXR expression had significantly increased after transfection with pSG5-hPXR expression plasmids, indicating that the PXR overexpression cells model was successfully established.  Figure 3(b) shows that significant increase of *CYP3A4* mRNA by RIF (as the positive control) was found to compare with the vehicle control (5.36-fold at 10 *μ*M, *P* < 0.01). At 10 *μ*M and 40 *μ*M, PC can significantly induce *CYP3A4* mRNA expression in transfected LS174T cells to 3.6- and 3.85-fold (*P* < 0.05, *P* < 0.01), respectively. However, in our previous study, we did not observe significant increase of *CYP3A4* mRNA level in the untransfected LS174T cells which was relatively low expression of PXR [28].

**Figure 4:**
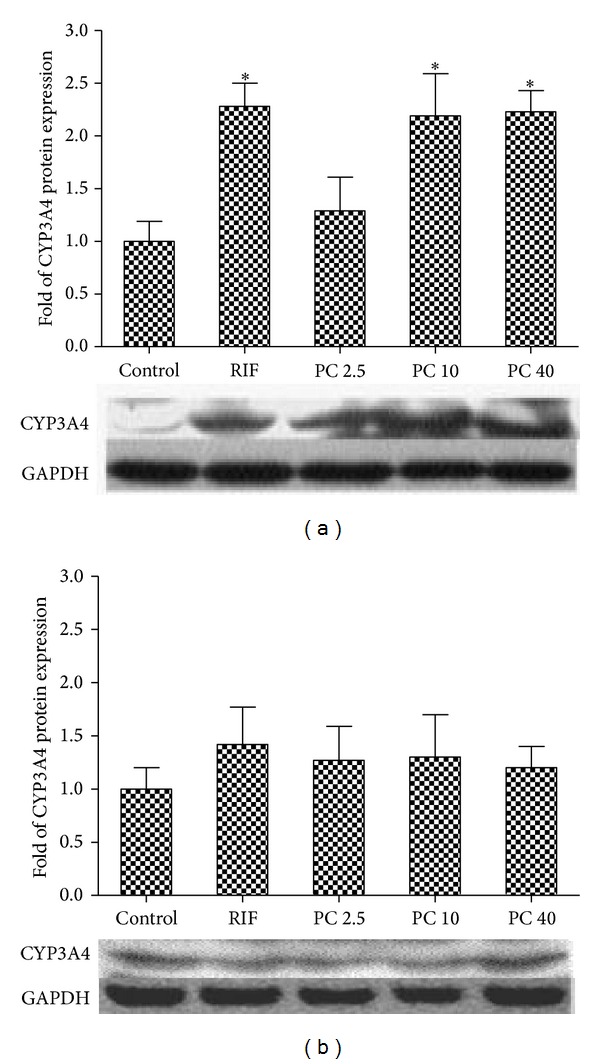
Effects of PC on the protein expression of CYP3A4 in LS174T cells. (a) LS174T cells were transfected with pSG5-hPXR expression plasmids for 6 h. The LS174T cells were treated with vehicle control (0.1% DMSO); 10 *μ*M RIF; and 2.5, 10, and 40 *μ*M PC for 72 h, respectively. The cell homogenates were subjected to western blot. (b) LS174T cells were untransfected with pSG5-hPXR expression plasmids for 6 h. The LS174T cells were treated with vehicle control (0.1% DMSO); 10 *μ*M RIF; and 2.5, 10, and 40 *μ*M PC for 72 h, respectively. The cell homogenates were subjected to western blot. The data were expressed as relative folds over vehicle controls. *P < 0.05 for comparison with the control groups. Values are expressed as mean ± S.E.M (*n* = 3).

To confirm that PC induces CYP3A4 through PXR-mediated pathway, CYP3A4 protein expression in PXR overexpressed LS174T cells were measured by Western blot analysis. After transfection with pSG5-hPXR expression plasmids, cells were exposed to PC at 2.5, 10, and 40 *μ*M for 72 h; CYP3A4 protein expression was subsequently measured by Western blot assay. As shown in Figure 4(a), LS174T cells transfected with PXR plasmid yielded significant increase to 2.28-fold (*P* < 0.05) in CYP3A4 protein expression after 72 h exposure to RIF at 10 *μ*M. Compared with the vehicle control, the expression was significantly induced to 2.19- and 2.22-fold by PC at 10 *μ*M and 40 *μ*M, respectively (*P* < 0.05). These results were generally consistent with those observed in the real-time PCR assay. Also, Figure 4(b) shows that the CYP3A4 protein expression was not increased by PC in untransfected LS174T cells.

**Figure 5:**
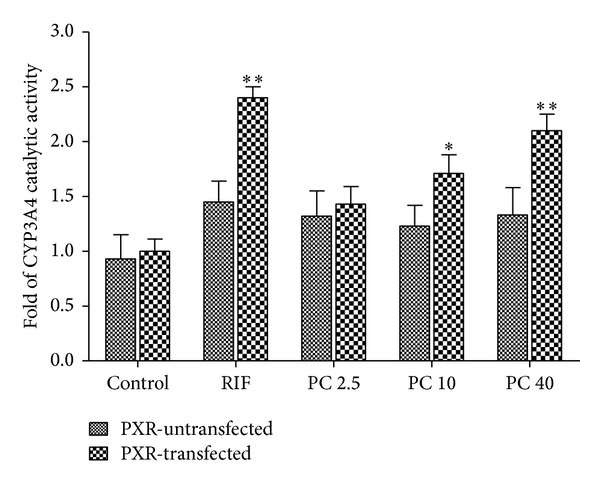
Determination of CYP3A4 catalytic activity. LS174T cells were transfected or untransfected with pSG5-hPXR expression plasmids for 6 h. The LS174T cells were treated with vehicle controls (0.1% DMSO); 10 *μ*M RIF; and 2.5, 10, and 40 *μ*M PC for 72 h, respectively. Total protein was isolated; total protein (1 mg/mL) was incubated with NIF and NADPH. The concentration of the DNIF, which is the metabolite of NIF through CYP3A4 pathway, was determined using the previously developed LC-MS/MS method. *P < 0.05, **P < 0.01, compared to control (DMSO) treatment group with PXR expression plasmid. Values are expressed as mean ± S.E.M (*n* = 3).

Furthermore, effect of PC on CYP3A4 enzyme activity was measured in PXR-overexpressed cells by LC-MS/MS assay. After transfection with PXR expression plasmids, cells were exposed to PC at 2.5, 10, and 40 *μ*M for 72 h; CYP3A4 activity was subsequently determined by LC-MS/MS assay based on measurement of specific CYP3A4-mediated Nifedipine dehydrogenation.  Figure 5 shows that LS174T cells transfected with plasmid encoding PXR plasmids yielded significant increase to 2.4-fold in CYP3A4 catalytic activity after 72 h exposure to RIF at 10 *μ*M (*P* < 0.01). LS174T cells transfected with plasmid encoding PXR plasmids yielded significant increase to 1.81- and 2.1-fold in CYP3A4 catalytic activity after 72 h exposure to PC at 10 *μ*M and 40 *μ*M, respectively, compared with control group (*P* < 0.05, *P* < 0.01). At the same time, we can also observe that CYP3A4 catalytic activity was not increased by PC in untransfected LS174T cells.

## 4. Discussion

As for the increasing wide use of herbal medicines, the potential of clinical herb-drug interactions are dramatically increased, which is frequently caused by induction or inhibition of metabolizing enzymes. CYP3A4 is the most abundant and important CYP isoform expressed in human liver. Important advances have been made in the understanding of the mechanisms involved in induction or inhibition of CYP3A4 [34]. Nuclear receptors, especially the PXR and CAR, are the most significant regulator of *CYP3A4* gene expression. Xenobiotics metabolism research has found that CYP3A4 expression and activity were affected by many ligands through both PXR and CAR pathways. PXR and CAR could generate the “cross talk” regulation effect on CYP3A4 transcription [35, 36].

Our previous results demonstrate that PC effectively transactivated mRNA expression, protein expression, and catalytic activity of CYP3A4 via the CAR-mediated pathway in human LS174T cells [27]. However, the effect of PC on the transactivation of CYP3A4 via PXR pathway is not fully understood, and it will be of clinical significance to elucidate the effect of PC on CYP3A4 through the PXR-mediated pathway, and then to find whether PC has “cross-talk” effect on PXR and CAR pathways. Therefore, in this study, we investigated the effect of PC on the *Cyp3a11* mRNA expression in PXR knockdown or PXR unknockdown mice primary hepatocytes, and regulations of CYP3A4 mRNA, protein expression, and catalytic activity by PC through PXR pathway were further investigated in human LS174T cells with overexpression of PXR.

Mice primary hepatocyte is an ideal model used for *PXR* mRNA knockdown for its higher endogenous PXR expression and easier obtain than human primary hepatocyte. PXR knockdown mice primary hepatocytes were successfully obtained by transient transfection of PXR siRNA and further validated by measuring the *PXR* mRNA level 72 h after transfection. We found that PC could significantly increase the level of *CYP3a11* gene expression in negative transfection groups, and the induction of *Cyp3a11* mRNA by PC was significantly suppressed in the *PXR* knockdown mice primary hepatocytes. These results demonstrate that knockdown of PXR suppresses the upregulation of *Cyp3a11* mRNA by PC, indicating that PXR pathway is causally involved as a contributing mediator.

On the other hand, LS174T cells has lower endogenous expression of PXR; thus, it is more ideal to transfect PXR into LS174T cells to produce the PXR high expression cell model. To confirm that PC induces CYP3A4 directly through PXR-mediated pathway, CYP3A4 mRNA, protein expression, and catalytic activity in PXR-overexpressed LS174T cells were measured by real-time PCR, western blot analysis, and LC-MS/MS assay. According to our previous results [28], *CYP3A4* mRNA expression could not be induced by PC in the LS174T cells without transfection with PXR plasmid. However, in the current study, after transfection with PXR plasmid, PC can significantly induce the level of *CYP3A4* mRNA. These results suggest that PC-mediated transactivation of *CYP3A4* gene via interaction with PXR pathway. Finally, we analyzed CYP3A4 protein expression and enzymatic activity in LS174T cells exposed to PA for 72 h after PXR transfection. Significant increase of CYP3A4 activity was observed in LS174T cells transfected with PXR plasmid. In order to further confirm the impacts of PC on the untransfected LS174T cells, the cells were treated with PC directly. The results show that increase of CYP3A4 protein expression could not be observed in untransfected LS174T cells, which was consistent with the results of mRNA expression and catalytic activity. The results indicate that PC can upregulate CYP3A4 protein level and its catalytic activity through PXR pathway.

As mentioned above, PXR and CAR are critical determinants of xenobiotics-induced CYP3A4 expression and they can generate “cross-talk” regulation on CYP3A4 transcription. It is not a surprise that compounds that regulate CYP3A4 via PXR pathway might also interact with CAR to activate CYP3A4. We recently reported that PC can effectively transactivate luciferase activity and mRNA and protein expression of CYP3A4 via CAR-mediated pathway in LS174T cells; the results demonstrate that CAR also plays a role in the activation of CYP3A4 by PC [27]. In current results, it was elucidated for the first time that PC can effectively transactivate CYP3A4 mRNA expression, protein expression, and catalytic activity via the PXR-mediated pathway in human LS174T cells. Combined with current results, PC can coactivate the CAR-mediated and PXR-mediated pathway to coregulate CYP3A expression. Therefore, PC could regulate CYP3A4 gene expression, protein expression, and activity through both PXR- and CAR-mediated pathways and thus accelerate detoxification and metabolism of CYP3A4 substrates. Further mechanistic studies are needed to investigate how the herbal compounds interact with PXR, CAR, and their coactivators and corepressors.

Besides PC, Praeruptorin A (PA) and Praeruptorin D (PD) are the other two major active constituents isolated from *Peucedanum praeruptorum* Dunn (Qianhu). For the increasing wide use of Qianhu and its active components such as PA, PC, and PD in the clinical practice, it is important to predict any potential herb-drug interactions between PA, PC, PD, Qianhu, and the other coadministered drugs. Most recently, we reported that PA and PD can also significantly upregulate CYP3A level via the PXR-mediated pathway [24, 25]. From the results of this study and our published data, we found that PD has the stronger regulation on CYP3A4 mRNA, protein, and enzyme activity via PXR pathway than PA and PC. However, there is no significant difference in the regulation of CYP3A by these three compounds. Very recently, we also found that PA and PC can significantly upregulate the expression of MRP2 and UGT1A1 via CAR-mediated pathway (data not shown). Taken together, all these findings suggest that PA, PC, and PD have varieties of effects on metabolizing enzymes or transporters via PXR or CAR mediated pathways; thus, this information should be taken into consideration to predict any potential herb-drug interactions between PA, PC, PD, *Peucedanum praeruptorum* Dunn, and the other coadministered drugs.

## 5. Conclusion

In summary, this study found that the level of *CYP3a11 *gene expression in mouse primary hepatocytes was significantly increased by PC, but such an induction was suppressed after knockdown of PXR by its siRNA. PC significantly enhanced CYP3A4 mRNA, protein expression, and functional activity through PXR-mediated pathway in PXR-overexpressed LS174T cells; conversely, no such increase was found in the untransfected cells. These findings suggest that PC can significantly upregulate CYP3A level via the PXR-mediated pathway and this should be taken into consideration to predict any potential herb-drug interactions between PC, Qianhu, and the other coadministered drugs. More attentions should be paid to ensure the safety in clinical utilization of Qianhu and PC.
